# GANsDTA: Predicting Drug-Target Binding Affinity Using GANs

**DOI:** 10.3389/fgene.2019.01243

**Published:** 2020-01-09

**Authors:** Lingling Zhao, Junjie Wang, Long Pang, Yang Liu, Jun Zhang

**Affiliations:** ^1^School of Computer Science and Technology, Harbin Institute of Technology, Harbin, China; ^2^Institute of Space Environment and Material Science, Harbin Institute of Technology, Harbin, China; ^3^Department of Rehabilitation, Heilongjiang Province Land Reclamation Headquarters General Hospital, Harbin, China

**Keywords:** drug-target affinity prediction, deep learning, semi-supervised, generative adversarial networks, convolutional neural networks

## Abstract

The computational prediction of interactions between drugs and targets is a standing challenge in drug discovery. State-of-the-art methods for drug-target interaction prediction are primarily based on supervised machine learning with known label information. However, in biomedicine, obtaining labeled training data is an expensive and a laborious process. This paper proposes a semi-supervised generative adversarial networks (GANs)-based method to predict binding affinity. Our method comprises two parts, two GANs for feature extraction and a regression network for prediction. The semi-supervised mechanism allows our model to learn proteins drugs features of both labeled and unlabeled data. We evaluate the performance of our method using multiple public datasets. Experimental results demonstrate that our method achieves competitive performance while utilizing freely available unlabeled data. Our results suggest that utilizing such unlabeled data can considerably help improve performance in various biomedical relation extraction processes, for example, Drug-Target interaction and protein-protein interaction, particularly when only limited labeled data are available in such tasks. To our best knowledge, this is the first semi-supervised GANs-based method to predict binding affinity.

## Introduction

A basic task in the field of new drug design and development is to model the interaction between known drugs and target proteins and to identify drugs with a high affinity for specific disease proteins (Cheng et al., 2018a; Cheng et al., 2019b). However, this is a rather challenging and expensive process even when only approximately 97M compounds reported by the PubChem database (Bolton et al., 2008) and 12K drug entries reported by the DrugBank (Wishart et al., 2006 are considered. Computational methods, especially machine learning models, can considerably accelerate the drug development process and save costs by guiding biological experiments.

Drug-target interaction (DTI) prediction (Yamanishi et al., 2010; Liu et al., 2016; Nascimento et al., 2016; Keum and Nam, 2017) was modeled as a binary classification problem and solved by a few traditional machine learning methods in recent decades. These methods have achieved remarkable performancehowever, they still exhibit limitations because of their strong dependence on handcrafted features.

Apart from predicting DTI, the drug-target binding afï- nity(DTA)(Pahikkala et al., 2014; He et al., 2017) attracts more interest as it can indicate the strength of the interaction between a DT pair. Therefore, predicting DTA can considerably benefit drug discovery, because the searching space would be narrowed down by pruning those DT pairs with low binding affinity scores. Kronecker regularized least squares (KronRLS) Pahikkala et al. (2014) and boosting machines (SimBoost) He et al. (2017) are two state-of-the-art methods for both DTI and DTA prediction. KronRLS is a similarity-based method and can predict the interaction by evaluating the structure similarity among compounds and targets. On the contrary, SimBoost utilizes a gradient boosting machine and belongs to feature-based methods; its feature involves similarity matrices of the drugs and those of targets He et al. (2017). The similarity-based methods (Cheng et al., 2018b) generally rely on similarities to predict the interaction of DT, which inevitably leads to bias. For the feature-based methods, more information regarding the DT are involved; but expert knowledge and feature engineering are also required to construct appropriate features.

Deep learning can represent and recognize the hidden patterns in the data well, therefore, deep-learning based methods have been proposed to predict DTI or DTA utilizing deep neural networks(DNN) (Peng-Wei et al., 2016; Tian et al., 2016; Hamanaka et al., 2017), convolutional neural networks(CNN), (Jastrzebski et al., 2016; Gomez-Bombarelli et al., 2018) recurrent neural networks(RNNs) and stacked-autoencoders based architectures. These methods facilitate the learning of the 3D structures provided and the bimolecular interaction mechanism. However, on one hand, this indeed improves the prediction as more important structural information is exploited, on the other hand, when the 3D structure is the input, these methods depend considerably on the availability of the known 3D structure of the protein-ligand complex.

Another deep-learning based method, called DeepDTA, was implemented to predict the binding affinities with CNN using only 1D representation, that is, the sequences of the proteins and simplified molecular input line entry system(SMILES)of the compounds. In DeepDTA, two CNN blocks are employed as feature extractors, and a fully connected layer receives the output of the CNN blocks and outputs the final prediction results. DeepDTA utilizes the strong representation of CNN, while avoiding the dependence on the 3D structure information, which results in remarkable performance over the other traditional machine learning methods. However, similar to all the state-of-the-art methods for DTA prediction, DeepDTA is also primarily based on supervised machine learning with known labels information. It is known that creating large sets of training data is prohibitively expensive and laborious, particularly in biomedicine, as domain knowledge is required.

An unsupervised learning method, generative adversarial networks(GANs), devised by Goodfellow et al. in 2014 (Goodfellow et al., 2014) may address the challenge. The GANs architecture is characterized by two differentiable functions that play different roles in refining the system. One differentiable function is known as a generator and the other as a discriminator. The generator learns to produce data from a learned probability distribution. The discriminator determines if the produced data is valid by determining if the input comes from the generator or from the actual data set. GANs and its variants have achieved great success in many applications such as computer vision and natural language processing. Additionally, GANs are more attractive as they can learn representations by reusing parts of the generator and discriminator networks as feature extractors, which can be widely applied in many supervised classification or prediction tasks. On the other hand, there also exist some problems in GANs, for example, the better the discriminator is, the more serious the gradient of the generator disappears; the adversarial network may cause the collapse of the model during training, this also brings inconvenience in the practical application. In order to solve these problems, researchers continue to push forward new improvement methods, including least squares GAN(LSGAN) Mao et al. (2017), Wasserstein GAN(WGAN) Arjovsky et al. (2017) conditional GAN(CGAN) Mirza and Osindero (2014), information maximizing GAN(infoGAN) Chen et al. (2016), energy-based GAN(EBGAN) Zhao and Mathieu. (2016), boundary-seeking GAN(BEGAN) Hjelm R D (2017) and so on.

Owing to the unsupervised characteristics of GANs, in this paper, we propose a GANs-based method to predict binding affinity, called GANsDTA for short. Our method comprises two types of networks, two partial GANs for the feature extraction from the raw protein sequences and SMILES strings separately and a regression network using convolutional neural networks for prediction. The contributions of this paper mainly include: We proposed a semi-supervised framework for DTA prediction; we adopted GAN to extract features of protein sequence and compound SMILES in an unsupervised way. Therefore, the proposed model can accommodate unlabeled data for the training as feature extractor using GANs does not require labeled data. This semi-supervised mechanism enables more datasets even without labels available for our model to learn proteins drugs features, leading to better feature representation and prediction performance accordingly. To our best knowledge, this is the first semi-supervised GAN-based method to predict binding affinity. Our results suggest that utilizing such unlabeled data can considerably help improve performance in various biomedical relation extraction processes, particularly when only limited labeled data (e.g. 2000 samples or less) is available in such tasks.

## Materials and Methods

### Data Sets

**Figure 1:**
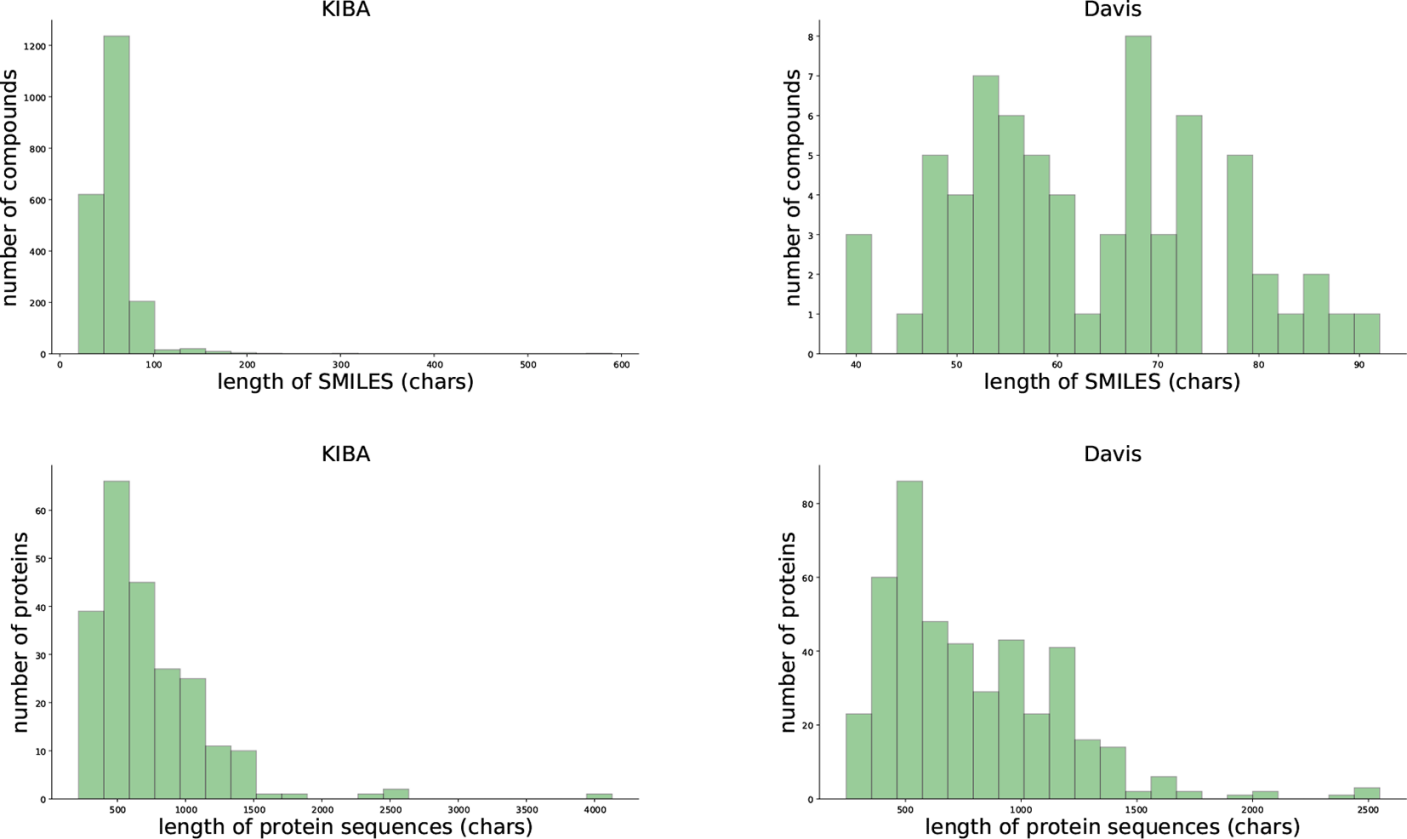
Summary of the KIBA (left panel) and Davis (right panel) data sets.

We evaluated our proposed method using two benchmark data sets, the Davis et al. (2011) and KIBA data set (Tang et al., 2014). **Table 1** and **Figure 1** provides the statistics of these two datasets.

**Table 1:** Data set.

	Proteins	Compounds	Interactions
Davis	442	68	30056
KIBA	229	2111	118254

### Proposed Method

#### Overview of our Approach

**Figure 2:**
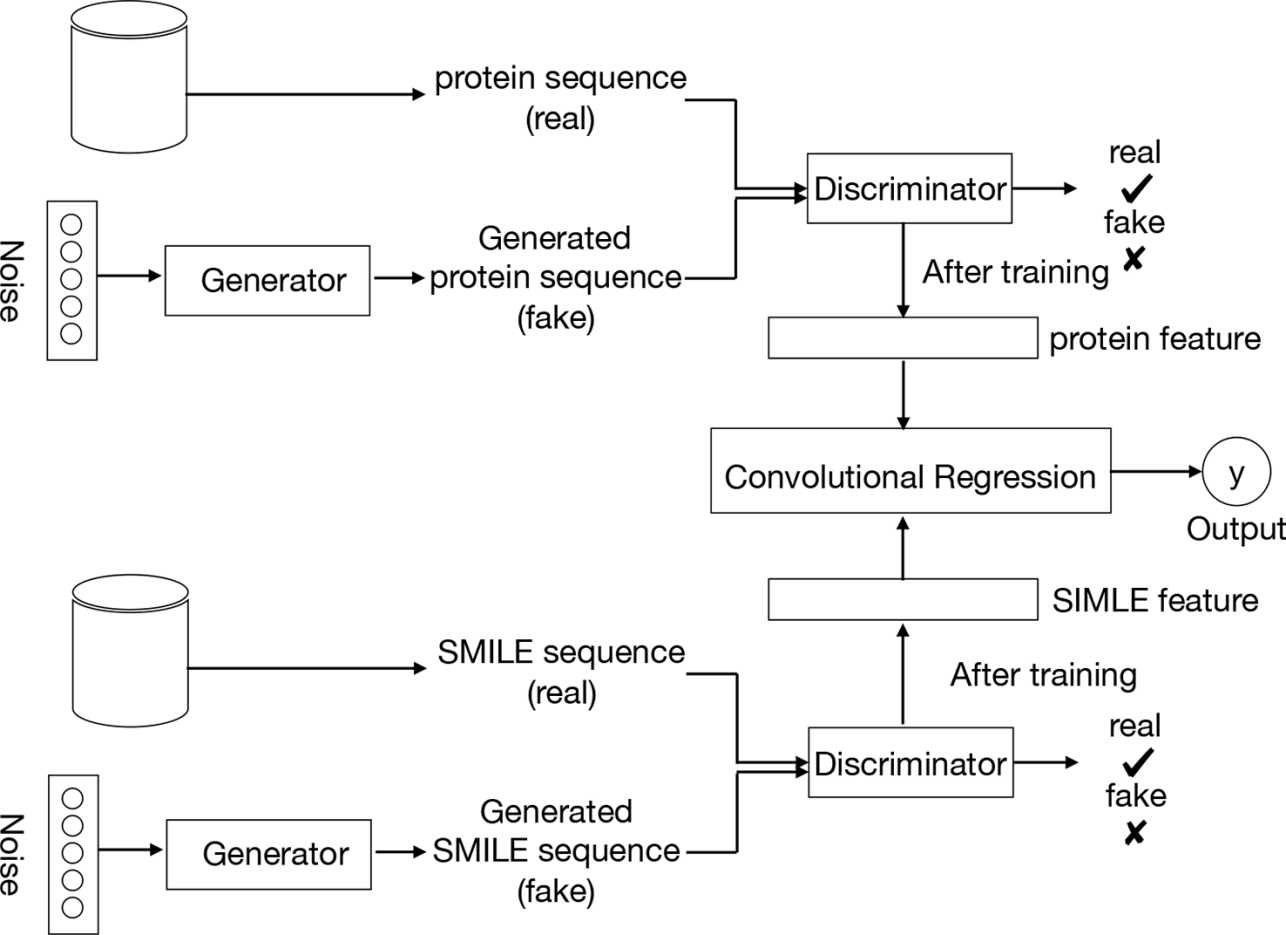
Pipeline overview. We train the GANs on the unlabeled data set. Compound SMILES and protein sequences are encoded and two independent GANs are applied to generate the fake samples. The trained discriminator of the GANs can then be used to project the labeled data sets into a feature latent space. Based on this feature, we train a convolutional regression to predict the DT binding affinity.

**Figure 2** provides an overview of the entire pipeline for our method for drug-target binding affinity prediction. Our approach comprises three elements: two feature extractors for protein sequence and compound, respectively, and a regressor for affinity value prediction. Each feature extractor is composed of a feature representation modular from GANs while the regressor is made up of a CNN. A two-round training pattern is employed. In the first training round, the feature extractors are trained in the context of GANs. First, fake samples are generated according to a given noise distribution by the generator of GANs, and then all the fake samples from the generator and the real samples from the available data sets are inputted to the discriminator network. In order to learn to distinguish real and fake sequences of proteins and SIMILES of compounds, the discriminator maps the input into a feature space by a local feature extractor, which promotes the sample classification. Thus, after the training of the whole GANs, a local feature extractor is obtained from the discriminator that can represent the characteristic of the input protein sequence or SMILE sequence. This trained local feature extractor is utilized as the feature representation of the proposed framework, followed by a regressor or classifier for prediction or classification task respectively. Finally, during the second round of training, with the labeled data (SIMILES and protein sequence) and fixed GANs-based feature extractor, the regressor is trained to minimize the loss function, leading to the optimal model parameters.

In the proposed method, the input proteins and drugs are treated as sequence representations. In particular, drugs are represented as SMILES strings – describing the chemical structure in short ASCII strings, and similarly, protein sequences are represented as a string of ASCII letters, which are the amino acids. Having the inputs as strings of text, the discriminator can learn the latent features of those sequences.

#### Feature Extracting Model

Goodfellow et al. (Goodfellow et al. (2014)) proposed a framework using a minimax game to train deep generative models, so called GANs. The GANs comprise two parts, a generator *G* and a discriminator *D*. The generator network *G* generates fake samples from the generator distribution *P_G_* by transforming a noise variable *z*∼*P_noise_*(*z*) into a sample *G*(*z*). The discriminators are to differentiate these generated samples following distribution *P_G_* from the true sample distribution *P_data_*. *G* and *D* are trained by playing against each other which can be formulated by a minimax game as follows:

(1) $$\underset{G}{\min}\underset{D}{\max}V(D,G)=E_{x\sim P_{data}}[\log(x)]+E_{z\sim Pnoise}[\log(1-D(G(z)))]$$

Meanwhile, for a given generator *G*, the optimal discriminator is *D*(*x*) = *P_data_*(*x*)/ (*P_data_*(*x*)+*P_G_*(*x*)).

**Figure 3:**
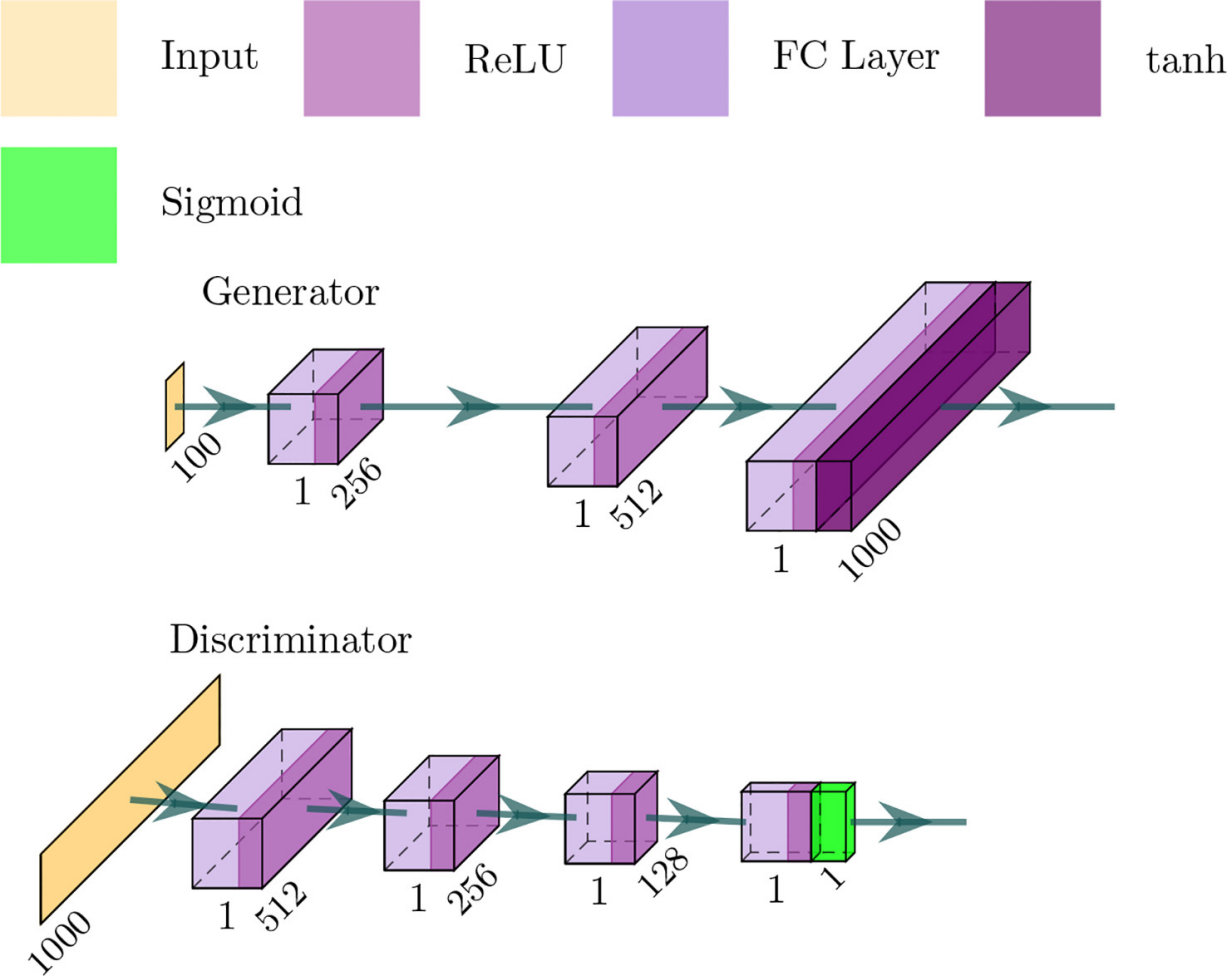
Architecture of the generator and discriminator networks in the proposed method.

The GANs employed in our framework is depicted in **Figure 3** — in which the generator network is a four-layer fully connected network and considers a noise vector as input — and produce a sequence of proteins or SMILES. The discriminator network is a three-layer fully connected network and the output is a probability value between 0 and 1, where 1 means that the input is real and 0 means that the input is fake.

Typically, the discriminator network can be decomposed into a feature extractor *F* (·;φ*_f_*) and a sigmoid classification layer with weight vector ψ*_l_*. Mathematically, given an input sequence *s*, we have

(2) $$D(s)=\text{sigmoid}(\phi_{l}^{T}F(s;\phi_{f}))=\text{sigmoid}(\phi_{l}^{T}f)$$

where ϕ= (ϕ*_f_*ϕ*_l_*) and *sigmoid*(*z*)=1/ (1+*e*^−z^). *f*=*F* (*s*;ϕ*_f_*) is the feature extractor of *s* in the last layer of *D*, which is to be leaked to the regression model.

### Regression Model

To predict the binding affinity, we combine the intermediate features learned by the two GANs and then apply a few 1D convolution layers to learn the final regression output. The convolution regression model conducts convolution operations with the kernel size of 4 to acquire feature maps of the input information. The dimension of the first convolution layer is 16×4. All the convolution layers are connected to activation functions (ReLU function). The dimensions of the second and third, convolution layers are 32×4, and 48×4. The activation function of the output layer is a linear function (identity function, i.e., *y = x*) that obtains a continuous value. This network is trained by minimizing the loss function defined by the mean square error (MSE) between the outputs *p* of this network and depth values *y* included in the dataset:

(3) $$MSE=\frac{1}{n}\sum_{k=1}^{n}{\left(p_{k}-y_{k}\right)}^{2}$$

## Experiments and Results

We compared our proposed method with the state-of-the-art DTA prediction models using the Davis and Kiba datasets. For these two datasets, we used the same setting as DeepDTA, that is, 80% of data were split as training samples and 20% as testing samples. In addition, our model is trained by both the labeled and unlabeled instances. We apply the Adam optimizer with the initial learning rate of 0.0001 to optimize the parameters of the model. We manually tuned the hyperparameters based on the testing results on the validation set. The performance of the proposed model was measured by calculating the concordance index (CI) and mean squared error (MSE) metrics. CI evaluates the ranking performance of the models that output continuous values.

(4) $$CI=\frac{1}{Z}\sum_{\delta_{x}>\delta_{y}}h(b_{x}-b_{y})$$

where *b_x_* is the prediction value for the larger affinity δ*_x_*, *b_y_* is the prediction value for the smaller affinity δ_y_, *Z* is a normalization constant, and *h*(*m*) is the step function.

(5) $$h(m)=\{\begin{array}{c} 1;\;ifm>0\\ 0.5;ifm=0\\ 0;\;ifm<0 \end{array}$$

MSE is a common measure to quantify the difference between the predicted values *p* and the actual values, which is defined as follows:

We compared the predicted performance of our method with DeepDTA and two machine-learning-based KronRLS and SimBoost method. Both of our work and DeepDTA only utilize the information of protein sequence and SMILES of the compounds. The difference is that our method can extract features of proteins and compounds in an unsupervised manner. **Tables 2** and **3** present the MSE and CI values for different methods for Davis and KIBA datasets.

**Table 2:** CI and MSE scores for the Davis dataset on the independent test for our method and other methods.

Method	Protein rep.	Compound rep.	CI	MSE
DeepDTA	Smith-Waterman	Pubchem-Sim	0.790	0.608
DeepDTA	Smith-Waterman	CNN	0.886	0.420
DeepDTA	CNN	Pubchem-Sim	0.835	0.419
DeepDTA	CNN	Pubchem-Sim	0.878	0.261
KronRLS	Smith-Waterman	Pubchem-Sim	0.871	0.379
SimBoost	Smith-Waterman	Pubchem-Sim	0.872	0.282
GANsDTA	GAN	GAN	**0.881**	0.276

Bolded texts mean the best results.

**Table 3:** CI and MSE scores for the Kiba dataset on the independent test.

Method	Protein rep.	Compound rep.	CI	MSE
DeepDTA	Smith-Waterman	Pubchem-Sim	0.710	0.502
DeepDTA	Smith-Waterman	CNN	0.854	0.204
DeepDTA	CNN	Pubchem-Sim	0.718	0.571
DeepDTA	CNN	CNN	0.863	0.194
KronRLS	Smith-Waterman	Pubchem-Sim	0.782	0.411
SimBoost	Smith-Waterman	Pubchem-Sim	0.836	0.222
GANsDTA	GAN	GAN	**0.866**	0.224

Bolded texts mean the best results.

For the Davis dataset (**Table 2**), even the DeepDTA, with Simith–Waterman as the protein’s representation form and drugs in the 1D strings, achieves the best CI score (0.886), slightly higher than our method - its MSE metric is much higher than our methods. Whereas another DeepDTA, CNN for protein and compound representation, achieves the best MSE with 0.261 as well as the lower CI than our method.

A similar performance is observed for the Kiba dataset (**Table 3**). In particular, DeepDTA is the best baseline in both measures, CI, at 0.863, and MSE, at 0.194, when both drugs and proteins are represented as ‘words’. Regarding CI, the proposed GANsDTA exhibits a slight improvement. The best CI GANsDTA gained is 0.866.

To provide a better assessment of our model, we determined the performances of GANsDTA, DeepDTA with two CNN modules and two baseline methods with two different metrics: $r_{m}^{2}$ index and area under precision recall (AUPR) score as well. $r_{m}^{2}$ index is a metric which defines the possibility of an acceptable model. Generally, if the value of $r_{m}^{2}$ the index is greater than 0.5 on a test set, we consider this model to be acceptable. The metric is described in equation (6) where r^2^ and *r*^0^ are the squared correlation coefficients with and without intercept, respectively. The details of the formulation are explained in Pratim Roy et al. (2009); Roy et al. (2013).

(6) $$r_{m}^{2}=r^{2}*(1-\sqrt{r^{2}-r_{0}^{2}})$$

The AUPR score is generally adopted for binary prediction. To measure AUPR based performances, the Davis and KIBA datasets should be converted into their binary forms *via* thresholding. For the Davis dataset we selected a pKd value of 7 as the threshold, while for KIBA dataset the threshold is 12.1, which is same as in the literature Öztürk et al. (2018).

**Tables 4** and **5** list the $r_{m}^{2}$ index and AUPR score of GANsDTA and three baseline methods on the Davis and KIBA datasets, respectively. The results suggest that SimBoost, DeepDTA and GANsDTA are acceptable models for to predict affinity with result to $r_{m}^{2}$ value.

**Table 4:** $r_{m}^{2}$ index and AUPR score for the Davis dataset.“4 $r_{m}^{2}$ index and AUPR score for the Davis dataset.”

Method	Protein rep.	Compound rep.	$r_{m}^{2}$	AUPR
DeepDTA	CNN	CNN	0.630	0.714
KronRLS	Smith-Waterman	Pubchem-Sim	0.407	0.661
SimBoost	Smith-Waterman	Pubchem-Sim	0.644	0.709
GANsDTA	GAN	GAN	0.653	0.691

**Table 5:** The $r_{m}^{2}$ index and AUPR score for the KIBA dataset.

Method	Protein rep.	Compound rep.	$r_{m}^{2}$	AUPR
DeepDTA	CNN	CNN	0.673	0.788
KronRLS	Smith-Waterman	Pubchem-Sim	0.342	0.635
SimBoost	Smith-Waterman	Pubchem-Sim	0.629	0.760
GANsDTA	GAN	GAN	0.675	0.753

**Figure 4:**
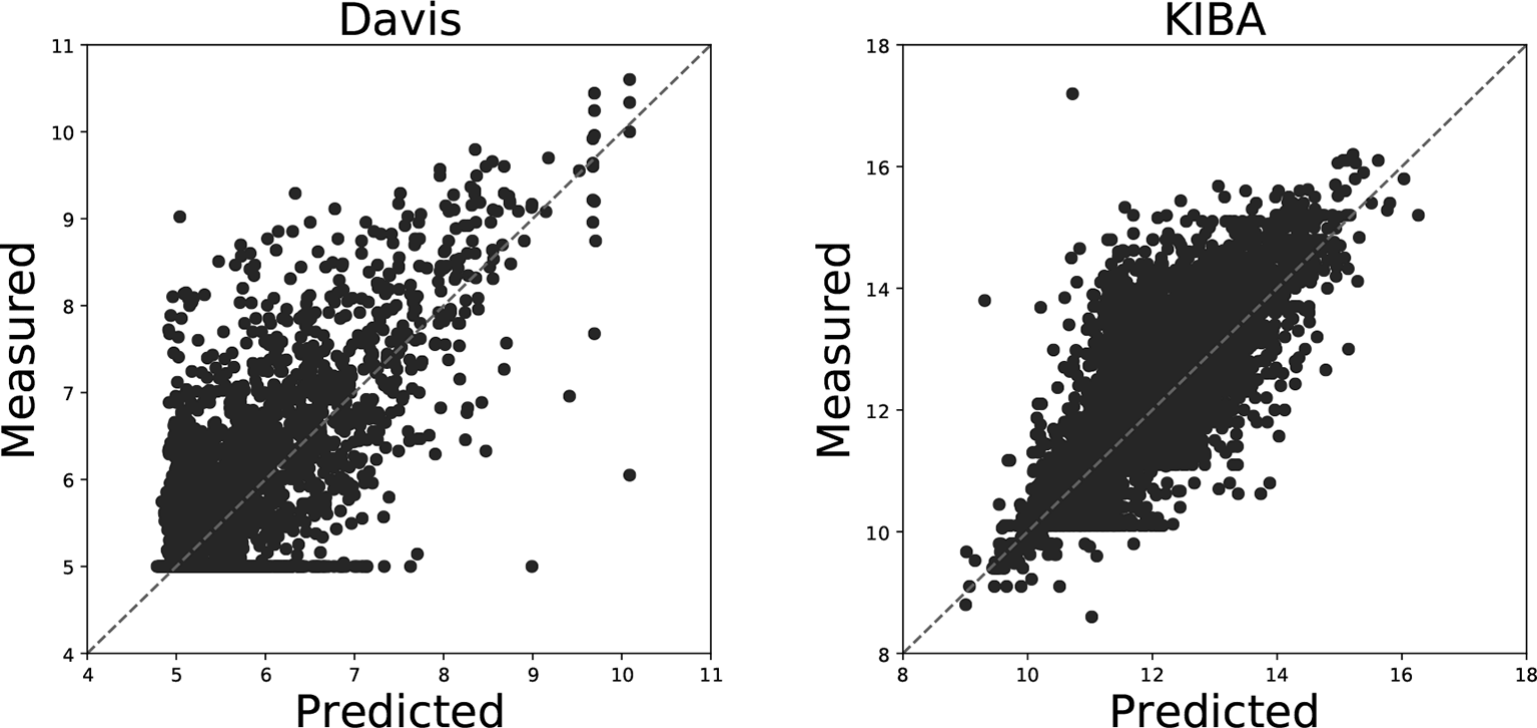
Predictions from DeepDTA model with two CNN blocks against measured (real) binding affinity values for Davis (pKd) and KIBA (KIBA score) datasets.

**Figure 4** illustrates the predicted binding affinity values against the actual values for our GANsDTA on the Davis and KIBA datasets. Evidently, an ideal model is expected to enable predictions (p) equal to the measured (y) values. For GANsDTA, it can be observed that the density is high around the *p = y* line, particularly for the KIBA dataset.

It can be observed that the proposed GANsDTA exhibits a similar performance to DeepDTA from **Tables 2**-**4**. For the Davis dataset, GANsDTA provides a slightly lower CI score (0.881) than the state-of-the-art DeepDTA with CNN the feature extraction (0.886), and a slightly higher MSE with 0.015. The reason is that the training for GANs is insufficient due to the small size of the Davis dataset which only includes 442 proteins, 68 compounds, and 30056 interactions. However, GANsDTA is still the second-best predictor. The other benchmark KIBA dataset includes 229 proteins, 2111 compounds, and 118254 interactions, enabling the GANs to be trained better, leading to better prediction accuracy. This indicates that GANsDTA is more suitable for the prediction task with a large dataset. In the future, more possible datasets (Cheng et al., 2018c; Cheng et al., 2019a) Cheng et al., 2016; Cheng et al., 2019a can be utilized to improve the training of GANsDTA.

## Conclusion

Predicting drug-target binding affinity is challenging in drug discovery. The supervised-based methods heavily depend on labeled data, which are expensive and difficult to obtain on a large scale. In this paper, we propose a semi-supervised GAN-based method to estimate drug-target binding affinity, while effectively learning useful features from both labeled and unlabeled data. We use GANs to learn representations from the raw sequence data of proteins and drugs and convolutional regression when predicting the affinity. We compare the performance of the proposed model with the state-of-art deep-learning-based method as our baseline. By utilizing the unlabeled data, our model can achieve competitive performance while using freely available unlabeled data. However, because it is difficult to train GANs, this approach is not comparative in the scenarios of a small dataset, and the improved techniques for training GANs should be employed to enhance the adaptability of GANs.

## Data Availability Statement

The datasets KIBA and Davis for this study can be found in http://www.ebi.ac.uk/biostudies/studies/S-EPMC6129291?xr=true.

## Author Contributions

LZ, JW, and LP substantially contributed to the conception and design of the study, and acquisition of data. YL analyzed and interpreted the data. LZ, JW, and JZ drafted the article.

## Funding

This work is supported by the National Natural Science Foundation of China (NSFC, Grant no.61305013 and 61872114).

## Conflict of Interest

The authors declare that the research was conducted without any commercial or financial relationships that could be construed as potential conflict of interest.

## References

[B1] Arjovsky M. Bottou L. Chintala S. (2017). Wasserstein generative adversarial networks. In International Conference on Machine Learning (ICML) 2017.

[B2] Bolton E. E. Thiessen. P. A. Wang Y. Bryant S. H. (2008). Pubchem: integrated platform of small molecules and biological activities. Annu. Rep. In Comput. Chem. 4, 217–241. 10.1016/s1574-1400(08)00012-1

[B3] Chen X. Houttooft R. Duan Y. (2016). Infogan: Interpretable representation learning by information maximizing generative adversarial nets. Adv. Neural Inf. Process. Syst. 2172–2180.

[B4] Cheng L. Sun J. Xu W. Dong L. Hu Y. Zhou M. (2016). Oahg: an integrated resource for annotating human genes with multi-level ontologies. Sci. Rep. 6, 34820. 10.1038/srep34820 27703231 PMC5050487

[B5] Cheng L. Hu Y. Sun J. Zhou M. Jiang Q. (2018a). Dincrna: a comprehensive web-based bioinformatics toolkit for exploring disease associations and ncrna function. Bioinformatics 34, 1953–, 1956. 10.1093/bioinformatics/bty002 29365045

[B6] Cheng L. Jiang Y. Ju H. Sun J. Peng J. Zhou M. (2018b). Infacront: calculating cross-ontology term similarities using information flow by a random walk. BMC Genomics 19, 919. 10.1186/s12864-017-4338-6 29363423 PMC5780854

[B7] Cheng L. Zhuang H. Yang S. Jiang H. Wang S. Zhang J. (2018c). Exposing the causal effect of c-reactive protein on the risk of type 2 diabetes mellitus: A mendelian randomization study. Front. Genet. 9, 657 =. 10.3389/fgene.2018.00657 30619477 PMC6306438

[B8] Cheng L. Wang P. Tian R. Wang S. Guo Q. Luo M. (2019). Lncrna2target v2.0: a comprehensive database for target genes of lncrnas in human and mouse. Nucleic Acids Res. 47, D140–D144. 10.1093/nar/gky1051 30380072 PMC6323902

[B9] Cheng L. Qi C. Zhuang H. Fu T. Zhang X. (2019a). gutmdisorder: a comprehensive database for dysbiosis of the gut microbiota in disorders and interventions. Nucleic Acids Res. 1–7. 10.1093/nar/gkz843 30629263 PMC6326781

[B10] Cheng L. Yang H. Zhao H. Pei X. Shi H. Sun J. (2019b). Metsigdis: a manually curated resource for the metabolic signatures of diseases. Brief Bioinform. 20, 203–209. 10.1093/bib/bbx103 28968812

[B11] Davis M. I. Hunt J. P. Herrgard S. Ciceri P. Wodicka L. M. Pallares G. (2011). Comprehensive analysis of kinase inhibitor selectivity. Nat. Biotechnol. 29, 1046. 10.1038/nbt.2017 22037378

[B12] Gómez-Bombarelli R. Wei J. N. Duvenaud D. Hernández-Lobato J. M. Sánchez-Lengeling B. Sheberla D. (2018). Automatic chemical design using a data-driven continuous representation of molecules. ACS Cent. Sci. 4, 268–276. 10.1021/acscentsci.7b00572 29532027 PMC5833007

[B13] Goodfellow I. Pouget-Abadie J. Mirza M. Xu B. Warde-Farley D. Ozair S. (2014). Generative adversarial nets. Adv. Neural Inf. Process. Syst. 2672–2680.

[B14] Hamanaka M. Taneishi K. Iwata H. Ye J. Pei J. Hou J. (2017). Cgbvs-dnn: Prediction of compound-protein interactions based on deep learning. Mol. Inf. 36, 1600045. 10.1002/minf.201600045 27515489

[B15] He T. Heidemeyer M. Ban F. Cherkasov A. Ester M. (2017). Simboost: a readacross approach for predicting drug-target binding affinities using gradient boosting machines. J. Cheminf. 9, 24. 10.1186/s13321-017-0209-z PMC5395521 29086119

[B16] Hjelm R. D. Che T. Jacob A. P. (2017). Boundary-seeking generative adversarial networks. arXiv preprint arXiv:1702.08431.

[B17] Jastrzebski S. Leśniak D. Czarnecki W. M. (2016). Learning to SMILE(S). In: International Conference on Learning Representation (Workshop track).

[B18] Keum J. Nam H. (2017). Self-blm: Prediction of drug-target interactions via self-training svm. PloS One 12, e0171839. 10.1371/journal.pone.0171839 28192537 PMC5305209

[B19] Liu Y. Wu M. Miao C. Zhao P. Li X.-L. (2016). Neighborhood regularized logistic matrix factorization for drug-target interaction prediction. PloS Comput. Biol. 12, e1004760. 10.1371/journal.pcbi.1004760 26872142 PMC4752318

[B20] Mao X. Xie H. Li Q. (2017). Least squares generative adversarial networks, in: Proceedings of the IEEE International Conference on Computer Vision, 2017 pp. 2794–2802. 10.1109/iccv.2017.304

[B21] Mirza M. Osindero. S. (2014). Conditional generative adversarial nets. arXiv preprint arXiv:1411.1784.

[B22] Nascimento A. C. Prudêncio R. B. Costa I. G. (2016). A multiple kernel learning algorithm for drug-target interaction prediction. BMC Bioinf. 17, 46. 10.1186/s12859-016-0890-3 PMC4722636 26801218

[B23] Öztürk H. Özgür A. Ozkirimli E. (2018). Deepdta: deep drug–target binding affinity prediction. Bioinformatics 34, i821–i829. 10.1093/bioinformatics/bty593 30423097 PMC6129291

[B24] Pahikkala T. Airola A. Pietilä S. Shakyawar S. Szwajda A. Tang J. (2014). Toward more realistic drug-target interaction predictions. Briefings In Bioinf. 16, 325–337. 10.1093/bib/bbu010 PMC4364066 24723570

[B25] Peng-Wei Chan K. C. You Z.-H. Chan K. C. C. You Z. H. (2016). “Large-scale prediction of drug-target interactions from deep representations,” International Joint Conference on Neural Networks (IJCNN) (IEEE), 1236–1243. 10.1109/ijcnn.2016.7727339

[B26] Pratim Roy P. Paul S. Mitra I. Roy K. (2009). On two novel parameters for validation of predictive qsar models. Molecules 14, 1660–, 1701. 10.3390/molecules14051660 19471190 PMC6254296

[B27] Roy K. Chakraborty P. Mitra I. Ojha P. K. Kar S. Das R. N. (2013). Some case studies on application of “rm2” metrics for judging quality of quantitative structure–activity relationship predictions: emphasis on scaling of response data. J. Comput. Chem. 34, 1071–, 1082. 10.1002/jcc.23231 23299630

[B28] Tang J. Szwajda A. Shakyawar S. Xu T. Hintsanen P. Wennerberg K. (2014). Making sense of large-scale kinase inhibitor bioactivity data sets: a comparative and integrative analysis. J. Chem. Inf. Modeling 54, 735–743. 10.1021/ci400709d 24521231

[B29] Tian K. Shao M. Wang Y. Guan J. Zhou S. (2016). Boosting compound-protein interaction prediction by deep learning. Methods 110, 64–72. 10.1016/j.ymeth.2016.06.024 27378654

[B30] Wishart D. S. Knox C. Guo A. C. Shrivastava S. Hassanali M. Stothard P. (2006). Drugbank: a comprehensive resource for in silico drug discovery and exploration. Nucleic Acids Res. 34, D668–D672. 10.1093/nar/gkj067 16381955 PMC1347430

[B31] Yamanishi Y. Kotera M. Kanehisa M. Goto S. (2010). Drug-target interaction prediction from chemical, genomic and pharmacological data in an integrated framework. Bioinformatics 26, i246–i254. 10.1093/bioinformatics/btq176 20529913 PMC2881361

[B32] Zhao J L. Y. Mathieu M. (2016). Energy-based generative adversarial network. arXiv preprint arXiv:1609.03126.

